# The Worsening of Heart Failure with Reduced Ejection Fraction: The Impact of the Number of Hospital Admissions in a Cohort of Patients

**DOI:** 10.3390/jcm12186082

**Published:** 2023-09-20

**Authors:** Jorge Perea-Armijo, José López-Aguilera, Rafael González-Manzanares, Cristina Pericet-Rodriguez, Juan Carlos Castillo-Domínguez, Gloria Heredia-Campos, Álvaro Roldán-Guerra, Cristina Urbano-Sánchez, Lucas Barreiro-Mesa, Nerea Aguayo-Caño, Mónica Delgado-Ortega, Manuel Crespín-Crespín, Martín Ruiz-Ortiz, Dolores Mesa-Rubio, Manuel Pan-Álvarez Osorio, Manuel Anguita-Sánchez

**Affiliations:** 1 Heart Failure Unit, Cardiology Departament, Reina Sofía University Hospital, Av. Menendez Pidal s/n, 14004 Cordoba, Spain; jorgeponde@hotmail.com (J.P.-A.); rafaelglezm@gmail.com (R.G.-M.); cristinapericetrodriguez@gmail.com (C.P.-R.); juanc.castillo.dominguez.sspa@juntadeandalucia.es (J.C.C.-D.); gmherediac@gmail.com (G.H.-C.); alvaroroldan97@gmail.com (Á.R.-G.); cristina97s.u@icloud.com (C.U.-S.); lucasbarme5@gmail.com (L.B.-M.); nereaguayo97@gmail.com (N.A.-C.); crespin2@hotmail.com (M.C.-C.); maruor@gmail.com (M.R.-O.); loladoctora@gmail.com (D.M.-R.); manuelpanalvarez@gmail.com (M.P.-Á.O.); manuelanguita@secardiologia.es (M.A.-S.); 2 Maimonides Institute for Biomedical Research of Cordoba, IMIBIC, 14004 Cordoba, Spain

**Keywords:** worsening of heart failure, reduced LVEF, hospital admission, prognosis, mortality

## Abstract

Background: Worsening heart failure (WFH) includes heart failure (HF) hospitalisation, representing a strong predictor of mortality in patients with heart failure with reduced ejection fraction (HFrEF). However, there is little evidence analysing the impact of the number of previous HF admissions. Our main objective was to analyse the clinical profile according to the number of previous admissions for HF and its prognostic impact in the medium and long term. Methods: A retrospective study of a cohort of patients with HFrEF, classified according to previous admissions: cohort-1 (0–1 previous admission) and cohort-2 (≥2 previous admissions). Clinical, echocardiographic and therapeutic variables were analysed, and the medium- and long-term impacts in terms of hospital readmissions and cardiovascular mortality were assessed. A total of 406 patients were analysed. Results: The mean age was 67.3 ± 12.6 years, with male predominance (73.9%). Some 88.9% (361 patients) were included in cohort-1, and 45 patients (11.1%) were included in cohort-2. Cohort-2 had a higher proportion of atrial fibrillation (49.9% vs. 73.3%; *p* = 0.003), chronic kidney disease (36.3% vs. 82.2%; *p* < 0.001), and anaemia (28.8% vs. 53.3%; *p* = 0.001). Despite having similar baseline ventricular structural parameters, cohort-1 showed better reverse remodelling. With a median follow-up of 60 months, cohort-1 had longer survival free of hospital readmissions for HF (37.5% vs. 92%; *p* < 0.001) and cardiovascular mortality (26.2% vs. 71.9%; *p* < 0.001), with differences from the first month. Conclusions: Patients with HFrEF and ≥2 previous admissions for HF have a higher proportion of comorbidities. These patients are associated with worse reverse remodelling and worse medium- and long-term prognoses from the early stages, wherein early identification is essential for close follow-up and optimal intensive treatment.

## 1. Introduction

Heart failure (HF) is a syndrome whose prevalence has been increasing in recent decades due to an increase in diagnoses and improved survival due to new therapeutic strategies [1]. Patients with heart failure with reduced ejection fraction (HFrEF) in the chronic phase are in a phase of apparent clinical stability, and despite pharmacological advances that have improved prognoses, these patients have a significant residual risk of clinical deterioration and mortality [1,2]. This risk increases several-fold if signs and symptoms coincide with a worsening of the disease, with worsening heart failure (WHF) defined as an increase in signs and symptoms of HF in patients with HFrEF despite optimal medical treatment [2]. Currently, this definition of WHF requires hospitalisation for HF, treatment of HF in the emergency department, or intravenous diuretics in the outpatient setting [2].

WHF can occur at any stage of disease progression, regardless of baseline LVEF [1,3]. In the different clinical trials of treatments with prognostic benefit in HFrEF, a prevalence of 15–30% of WHF has been observed during follow-up [4,5,6], which is consistent with the findings of other studies [1]. It has been observed that patients with WHF have a higher prevalence of different comorbidities (chronic kidney disease (CKD), diabetes mellitus, and atrial fibrillation (AF), among others) and, in addition, WHF is a strong predictor of mortality [7].

In this way, those patients with HFrEF who develop a WHF during follow-up should be considered at very high risk [4]. However, there is little evidence analysing the prognostic impact of the number of previous admissions for HF. For this reason, it is important to analyse those patients with HFrEF with a higher number of previous admissions in order to know their prognostic impact, as well as to analyse the clinical profile of these patients in order to identify early those patients with a higher risk of rehospitalisation for HF, and therefore a higher risk of mortality.

Thus, the main objective of the study is to analyse the impact of the number of previous HF admissions on the medium-term prognosis in terms of cardiovascular mortality and risk of hospital readmission for HF in a cohort of patients with HFrEF. The other objectives of the present study are as follows: to establish the differential clinical characteristics of a population with ≥2 previous HF admissions; to study the optimisation of treatment of prognostic benefit in HFrEF; and to analyse its impact on ventricular remodelling, neurohormonal response, and hospital readmissions for HF.

## 2. Material and Methods

### 2.1. Design and Study Population

**Figure 1:**
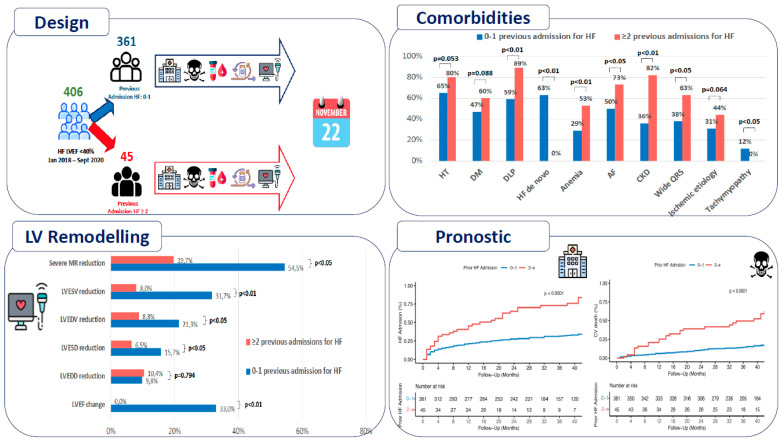
This figure shows the study design and the main results of comorbidities, left ventricular remodelling, and the prognosis of both cohorts. In blue are the results of cohort 1, and in red are the results of cohort 2.

This is an observational, retrospective and analytical study of real clinical practice, in which all patients with a diagnosis of HFrEF who were consecutively seen after hospitalisation or consultation for symptoms of HF in our hospital from January 2018 to September 2020 were included; follow-up ended in November 2022 for the occurrence of new admissions for HF or cardiovascular mortality. Patients who underwent heart transplantation during follow-up were excluded from the analysis, as shown in Figure 1.

A total of 409 patients were included and divided into two cohorts according to the number of HF admissions prior to the start of follow-up: *Cohort 1* included patients with 0–1 prior admission; *Cohort 2* included patients with ≥2 prior admissions, Figure S1.

### 2.2. Clinical and Analytical Variables

The aetiology of HF was established according to clinical criteria and the results of the complementary tests performed. HF symptoms were defined according to New York Heart Association (NYHA) functional class. Previous admission for HF was defined as all admissions for HF requiring increased intravenous diuretic and/or inotropic treatment from diagnosis of HFrEF until the start of follow-up. Disease evolution was defined as the time elapsed from the patient’s diagnosis of HFrEF until the start of follow-up in our study. Comorbidities were established according to medical history data at the start of follow-up.

Baseline and end-of-follow-up laboratory parameters were analysed, and anaemia was defined as the presence of anaemia when haemoglobin levels were <13 g/dL in men and <12 g/dL in women. CKD was assigned if it met the definition of the KDIGO guidelines [8]. A wide QRS was defined as a QRS with a duration of ≥120 ms.

Treatment-related data were analysed at baseline and at the end of follow-up. We recorded the presence of treatments indicated for HFrEF, such as the use of renin angiotensin axis inhibitors or antagonists (ACE inhibitors or ARB), angiotensin-neprilysin receptor inhibitors (ARNI), β-blockers (βB), mineralocorticoid receptor antagonists (MRA), ivabradine, loop diuretics, thiazides, sodium-glucose cotransporter 2 inhibitors (SGLT2i), cardiac resynchronisation therapy (defined as resynchronisation therapy (CRT) implantation and physiological His bundle or left bundle branch pacing), implantable cardioverter defibrillator (ICD), and percutaneous treatment of mitral regurgitation with an edge-to-edge approach device.

### 2.3. Echocardiographic Variables

Data from the baseline echocardiogram and the last echocardiogram performed during follow-up were analysed. The variables of interest were left ventricular end-diastolic volume (LVEDV) (mL), left ventricular end-systolic volume (LVESV) (mL), left ventricular end-diastolic diameter (LVEDD) (mm), left ventricular end-systolic diameter (LVEDD), (mm), and LVEF (%), determined via the Teicholz or Simpson method.

### 2.4. Outcome Variables

Prognosis was assessed by the incidence of cardiovascular mortality or hospital readmissions for heart failure.

### 2.5. Statistical Analysis

Quantitative variables were expressed as mean and standard deviation (if the distribution of values did not conform to normality, median and interquartile range); categorical variables were expressed as absolute frequencies and percentages. The normality of quantitative variables was studied using the Shapiro–Wilk test. To compare the variables between the two groups, appropriate parametric and non-parametric tests were used (Chi-square or Fisher’s exact test for qualitative variables, and Student’s *t*-test or Mann–Whitney U-test for quantitative variables). To compare changes in qualitative and quantitative variables in the same group over time, McNemar and Wilcoxon tests were used, respectively. Event-free survival (HF readmissions, cardiovascular mortality) in both groups was studied using the Kaplan–Meier method, and differences between the survival curves of the two groups were analysed using the Log-Rank test. In addition, the survival study was completed via a univariate and multivariate Cox regression analysis. In the multivariable model, collinearity was taken into account, and a stepwise selection method with backward elimination was used, initially including those variables considered clinically relevant and those with *p* < 0.100 in the univariable analysis. All contrasts were bilateral, and those wherein *p* < 0.05 were considered significant. Data were collected, processed, and analysed using SPSS version 25 (IBM Corporation, Amonk, NY, USA) and R 4.2.1 (The R Foundation, Viena, Austria).

### 2.6. Ethical Considerations

The study was approved by the Cordoba provincial research ethics committee with committee reference 5202, with a favourable opinion given on 27 October 2021.

The study was subject to the standards of good clinical practice and complied at all times with the ethical precepts contained in the Declaration of Helsinki with its latest updates, including the Oviedo agreement. The confidentiality of the data was respected at all times, through the anonymity of the data in the database in accordance with Royal Decree 1720/2007, which implements Organic Law 15/1999 of 13 December, on Personal Data Protection.

## 3. Results

### 3.1. Clinical Characteristics

A total of 409 patients diagnosed with HFrEF were studied, of whom 3 could not be classified due to lack of data, with a mean age of 67.3 ± 12.6 years, and 79.3% (300 patients) were male. Of the total, 361 patients (88.9%) were included in ***Cohort 1***, and 45 (11.1%) in ***Cohort 2***. Baseline characteristics of both groups are shown in Table 1. ***Cohort 1*** had a higher proportion of de novo HF (62.6% vs. 0%; *p* < 0.001) and a shorter disease evolution time (28.0 ± 60.3 vs. 103.6 ± 77.9 months; *p* < 0.001). ***Cohort 2*** had a higher proportion of wide QRS (37.8% vs. 63.2%; *p* = 0.003), as well as a higher proportion of comorbidities such as dyslipidaemia (58.9% vs. 88.9%; *p* < 0.001), AF (49.9% vs. 73.3%; *p* = 0.003), and CKD (36.3% vs. 82.2%; *p* < 0.001), with a higher proportion of more advanced stages and anaemia (28.8% vs. 53.3%; *p* = 0.001). There were no significant differences in the remaining parameters. At the end of follow-up, ***cohort 2*** had a worse NYHA functional of class III–IV (15.8% vs. 60%; *p* < 0.001).

### 3.2. Aetiology of HF

In our cohort of patients, the most frequent cause of HF was ischaemic, with 131 patients (32.3%), followed by idiopathic, with 125 patients (30.8%). ***Cohort 1*** had a higher proportion of tachycardiomyopathy (12.2% vs. 0%; *p* = 0.009) and a non-significant lower trend of ischaemic aetiology compared to ***cohort 2*** (30.7% vs. 44.4%; *p* = 0.064), Table 1.

### 3.3. Echocardiographic Parameters

Regarding the structural study of the left ventricle, ***cohort 1*** had lower LVEDD at baseline (61.0 [57–67] vs. 67.0 [58.8–70.3] mm; *p* = 0.032), with no differences in baseline LVEF data. ***Cohort 1*** had better reverse remodelling at the end of follow-up, with increased LVEF (30.0% [IQR 26–35] vs. 40.0% [IQR 31–53.5]; *p* < 0.001) and reduced ventricular diameters [LVEDD (61.0 [IQR 57–67] vs. 58.0 [IQR 52.0–62.5]; *p* = 0.01), LVESD (51.0 [IQR 47–59] vs. 43.0 [IQR 37–51.3]; *p* < 0.001)], as well as reduced volumes [LVEDV (150.0 [IQR 117–183] vs. 118 [IQR 87.5–164]; *p* < 0.001) and LVESV (101.0 [IQR 78–130.8] vs. 69 [IQR 45–105.3]; *p* < 0.001)]; however, no such left ventricular remodelling was observed in ***cohort 2***, with only a significant reduction in LVEDD at the end of follow-up (65.3 ± 8.2 vs. 60.8 ± 6.6 mm, *p* = 0.01), with no change in LVEF or ventricular volumes, as shown in Table 2. Regarding the prevalence of severe functional mitral regurgitation, with no differences at baseline, there was a higher proportion in cohort 2 at the end of the follow-up period (4.6% vs. 14.3%; *p* = 0.004), as shown in Table 2. In our cardiac imaging laboratory, the limits of agreement for EF measurements through the Simpson biplane method were −5% to 5.3% (inter-observer) and −6% to 5.8% (intra-observer) [9].

### 3.4. Neurohormonal Response

On analysis of neurohormonal response, ***cohort 1*** had lower baseline NT-proBNP (4640.0 [IQR 1873–10,431] vs. 6297.0 [IQR 3981.8–18,075.8]; *p* = 0.005), with no difference in baseline CA125 levels. At the end of follow-up, ***cohort 1*** showed a significant reduction in both NT-proBNP levels (4640.0 [IQR 1873.0–10,431.0] vs. 1599.0 [IQR 522.8–5148.5]; *p* < 0.001) and CA125 (19.8 [IQR 9.4–65.8] vs. 10.1 [6.2–20.9]; *p* = 0.011), with no reduction in neurohormonal responses observed in ***cohort 2***, as shown in Table 2.

### 3.5. Optimising Treatment of HFrEF

At baseline follow-up, ***cohort 1*** had a higher prescription of ARBs (59.3% vs. 42.2%; *p* = 0.029), while ***cohort 2*** had a higher prescription of diuretics, both loop diuretics (78.4% vs. 95.6%; *p* = 0.005) and thiazides (16.3% vs. 35.6%; *p* = 0.004); ***Cohort 2*** also had a higher indication for devices at baseline, both ICDs (6.9% vs. 22.2%; *p* = 0.001) and cardiac resynchronisation (2.5% vs. 22.2%; *p* < 0.001). At the end of follow-up, ***cohort 2*** persisted in a higher prescription of diuretics, both loop diuretics (68.7% vs. 95.6%; *p* < 0.001) and thiazides (15.5% vs. 46.7%; *p* < 0.001), ICDs (15.2% vs. 35.6%, *p* = 0.001) and resynchronisation therapy (14.7% vs. 33.3%; *p* = 0.002), Table 2. In addition, higher doses of loop diuretics (3% vs. 8.9%; *p* < 0.001) and thiazides (3% vs. 17.8%; *p* < 0.001) were also observed in ***cohort 2.*** No differences were found in the remaining treatment.

### 3.6. Prognosis: HF Readmissions and Cardiovascular Mortality

**Figure 2:**
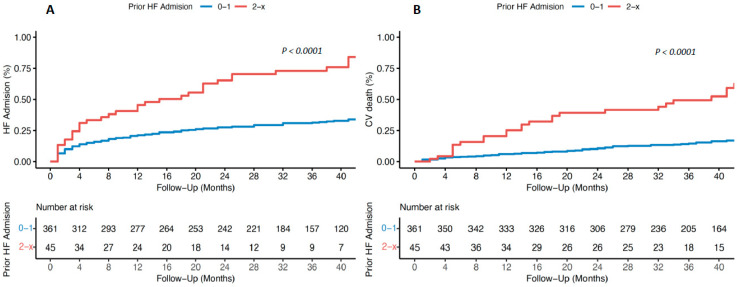
Kaplan–Meier curve of time to readmission for heart failure (**A**) and cardiovascular mortality (**B**) in patients with heart failure and a history of ≤ 1 and ≥2 admissions for HF.

The overall series had a median follow-up of 37.0 months (IQR 27.0–45.3 months). *Cohort 1* had a higher HF readmission-free survival at 1 year (21.3% vs. 40.5%; *p* < 0.001) and 5-year follow-up (37.5% vs. 92%; *p* < 0.001) with differences emerging from the start of the follow-up, Figure 2. The number of patients who died in the follow-up were 123 (30.5%), 90 of them due to HF and 33 of them due to non-cardiovascular pathology, Table S1. *Cohort 1* had lower cardiovascular mortality at 12 months (8.0% vs. 24.4%; *p* < 0.001) and at 60 months follow-up (36.5% vs. 76.0%; *p* < 0.001), Figure 2.

### 3.7. Multivariate Analysis: Predictor Variables for Mortality and HF Readmission

Different variables that could influence cardiovascular mortality and HF readmission were included. Those variables statistically significant in the univariate analysis were included in the multivariate analysis. Independent predictors of HF readmission, according to our model, were >1 previous HF admissions [HR 2.11, 95%CI 1.30–3.21, *p* < 0.001], CKD [HR 2.52, 95%CI 1.66–3.82, *p* < 0.001], anaemia [HR 1.86, 95%CI 1.27–2.74, *p* = 0.002], and baseline NYHA III–IV [HR 2.18, 95%CI 1.50–3.17, *p* < 0.001], as shown in Table 3. For cardiovascular mortality, independent predictors were age [HR 1.04, 95%CI 1.01–1.06, *p* = 0.004], >1 previous HF admissions [HR 2.84, 95%CI 1.75–4.59, *p* < 0.001], CKD [HR 2.38, 95%CI 1.39–4.06, *p* = 0.001], anaemia [HR 2.01, 95%CI 1.23–3.28, *p* = 0.005], and baseline NYHA III–IV [HR 2.54, 95%CI 1.61–4.02, *p* < 0.001], Table 3. On the other hand, obesity behaved as a protective factor for hospital readmission for heart failure [HR 0.71, 95%CI 0.57–0.88, *p* = 0.002].

## 4. Discussion

### 4.1. Selection, Classification and Clinical Characteristics of Patients with ≥2 Previous Admissions for HF

The present study analyses the prognostic impact of the number of HF admissions prior to the start of follow-up in a cohort of 406 patients with HFrEF. Previous studies have analysed the impact of HF hospitalisations at follow-up [1,7,10] and the impact of an admission in the year prior to the start of follow-up [11,12,13], both of which showed a worse prognosis. However, in the present study, the analysis focused on the impact of the number of previous HF admissions since the diagnosis of the cardiac condition. We believe that this analysis is more similar to routine clinical practice, allowing us to know how the disease has evolved since the beginning of the diagnosis in each patient.

In our population, 11% of the patients had had ≥2 admissions for HF. The observed prevalence of WHF varies between studies, but ranges from 15–30% [1,4,5,6], while the identified prevalence of patients with recent admissions is around 15–20% [10,13]. In our case, the fact that 11% of patients have had ≥2 previous admissions for HF indicates that a not insignificant percentage have had several previous WHF and represent a highly complex and high-risk subgroup.

Patients with ≥2 previous admissions for HF appear to have a different clinical profile, with a lower proportion of de novo HF and, logically, a longer disease course. Furthermore, there were no differences in baseline NYHA functional class, suggesting that despite having had more previous admissions for HF, they were not clinically worse at the start of follow-up, which could be related to the ‘saw-tooth’ pattern of disease progression and subsequent improvement after decompensation that many patients experience [14]. However, as more readmissions occur, there is a clear worsening of functional class, which may correspond to more advanced stages of disease where poor functional class persists despite improvement after hospital admission.

In addition, a higher proportion of dyslipidaemia, AF, wide QRS, CKD, anaemia and a lower prevalence of tachycardiomopathy were identified in patients with ≥2 previous admissions. These findings are consistent with those observed in other studies which also identify that patients who have been admitted more often have a higher proportion of comorbidities such as AF, CKD and anaemia [10,13], many of which have been associated with more hospitalisations and higher HF mortality [13].

### 4.2. Neurohormonal Response

In our population, it has been observed that patients with 0–1 previous admissions had higher baseline NT-proBNP levels as observed in previous studies [3,15]. However, no differences in baseline CA125 levels were found in our population, despite the fact that this marker has also been associated with a higher rate of readmission for HF and mortality [16].

Furthermore, the group of patients with 0–1 previous readmissions showed a significant reduction in both NT-proBNP and CA125 levels, which could be related to an improvement in congestion and, as a result, a reduced need for diuretics and fewer rehospitalisations for HF. This biomarker evolution could be useful in guiding follow-up and treatment, as has been shown in other studies with favourable results [2,17]. Like CA125, other biomarkers such as BNP, estimated plasma volume status (ePVS), hydration status assessed by bioimpedance (BIVA) and blood urea nitrogen/creatinine ratio (BUN/Cr) have been shown to predict the prognosis of heart failure patients regardless of acute or chronic heart failure status. Thus, they all allow the identification of patients with higher congestion and worse prognosis, and could be tools to help guide depletive therapy [18].

### 4.3. Analysis of Cardiac Remodelling

Regarding structural analysis, those patients with ≥2 previous admission for HF have a larger baseline diastolic diameter and volume, suggesting, together with other data discussed above, that the disease in this subgroup is more advanced. Regarding the analysis of ventricular function, both groups have a similar baseline LVEF, although there are disparate results in previous studies because some authors observe a greater tendency for WHF in those with lower baseline LVEF, even within HFrEF [19], while other studies suggest that the worse prognosis is independent of baseline LVEF [1,3].

In relation to cardiac remodelling, greater reverse remodelling and improvement in LVEF according to the criteria of the Universal Definition of HF 2021 [20] have been observed in the group with fewer previous admissions. This group of patients has similar characteristics to those in other studies assessing the prognostic impact of LVEF improvement using the same criteria used in this study. These characteristics include a lower prevalence of comorbidities, less dilated left ventricles and shorter HF evolution time [21].

The structural changes observed may be influenced by the better optimisation of treatment that has occurred in the subgroup with fewer admissions in our series (greater increase in the prescription of resynchronisation therapy and ARNI), since these are two therapies that are associated with reverse remodelling and improvement in LVEF, evaluated in the REVERSE [22] and PROVE-HF [23] studies, respectively. However, in the published studies on WHF, these data on changes in ventricular remodelling have not been evaluated, and furthermore, data on LVEF improvement are unknown [1,3,7,13].

### 4.4. Optimising Treatment of HFrEF

Regarding treatment with prognostic benefit of HFrEF, at baseline, there was a lower proportion of ARBs in those with ≥2 previous admissions, probably due to a non-significantly higher prescription of ARNI and/or higher intolerance due to hypotension. At follow-up, there was a slight reduction in the prescription of β-blockers in both groups, which may be related to intolerance to β-blockers. In addition, there was a higher proportion of ICD/CRT in this group, probably because they were patients with longer disease evolution, more prevalence of ischaemic aetiology, greater persistence of left ventricular dysfunction, and a greater proportion of wide QRS.

In relation to treatment at the end of follow-up, although there was a lower proportion of improvement in the group ≥2 prior admissions, except for the higher proportion of CRT in this group, no significant differences were observed in drug treatment of prognostic benefit in HFrEF, similar to what was observed by Mallick et al. [3]. In this regard, in some studies assessing the prognostic impact of LVEF improvement, it has also been observed that no differences were found in drug treatment with prognostic benefit for HFrEF, except in SGLT2i, regardless of LVEF improvement during follow-up [21]. This may be due to the fact that LVEF recovery does not necessarily imply complete remission of the disease, as a biochemical profile of neurohormonal activation has been found to persist, indicating that there is still a risk of cardiovascular events. Therefore, discontinuation of treatment is not recommended, as this is associated with an increased likelihood of recurrence of dysfunction, and increased hospital readmissions for HF [24].

Medical treatment was generally well optimised, except for a low rate of introduction of SGLT2i, probably due to the fact that this pharmacological group was not recommended for use from the start in non-diabetic HFrEF until the publication of the ESC HF guidelines in 2021 [25]. On the other hand, we observed a greater deprescription of loop diuretics in the group of patients with fewer admissions compared with the other group, a finding also reported in other studies [3]. This could be related to a worse functional class, higher NT-proBNP levels, and a higher rate of hospital readmissions for HF in patients with HF and a higher number of previous admissions.

### 4.5. Prognostic Impact and Independent Prognostic Factors

In our study, CKD and anaemia were found to be independent factors for higher HF readmission rates and cardiovascular mortality, as observed in other studies [10,13]. However, in relation to anaemia, another study has observed that anaemia is related to renal function and biomarkers of central and peripheral congestion, which have a predictive role in the rate of all-cause mortality in heart failure patients, unlike anaemia [26]. Thus, although anaemia has been associated with higher rates of HF hospitalisations and mortality, some studies suggest that it may be due to other factors associated with anaemia, rather than anaemia per se.

Obesity has also been found to be associated with a lower rate of heart failure admissions. However, this finding could be confounded by sarcopenia and cardiac cachexia in patients with more advanced HF, the presence of which is associated with a worse prognosis [27], and therefore obesity would not represent a clear protective factor.

In terms of prognostic impact, those with ≥2 previous admissions were associated with a worse prognosis in terms of rehospitalisations for HF and cardiovascular mortality, it also being an independent predictor of both cardiovascular outcomes. These differences are observed from the first months of follow-up, and are consistent with the findings of other studies [1,3,7,11,13]. Furthermore, it should be noted that this group has a very poor prognosis, with nearly 50% having died of HF at 3 years of follow-up, and nearly 75% at 5 years despite optimal medical treatment, which gives an idea of the severity associated with these patients, making early identification, close follow-up, and early intensive treatment of this subgroup of patients essential.

Moreover, it would be desirable to optimise treatment in these patients, as underuse of pharmacological treatment with prognostic benefit has been observed after HF worsening [28], and it is also essential to develop new therapeutic options in this high-risk HF group to improve their prognosis. In this regard, the VICTORIA trial evaluated the impact of vericiguat in patients with HFrEF with evidence of prior WHF, a subgroup of patients with 41% NYHA functional class III–IV and higher baseline NT-proBNP levels than others recently published clinical trials. In this clinical trial, the addition of vericiguat resulted in a reduction in cardiovascular mortality or HF hospitalisation after 3 months of treatment with this guanylate cyclase stimulator [6]. In addition, patients with a WHF in the previous 3 months were associated with a higher risk of events, and these patients also had a reduction in these events [12]. Other studies such as the GALATIC-HF trial evaluated Omecamtiv Mecarbil in patients with HFrEF with current or recent (<1 year) admission for HF, with a percentage of NYHA functional class III–IV and NT-proBNP levels similar to VICTORIA-HF. A reduction in HF events or deaths from cardiovascular causes was observed in the Omecamtiv group [29], so this could be a therapeutic alternative in these high-risk patients.

### 4.6. Limitations

The study is single-centre and retrospective. A prospective design would eliminate the potential selection bias associated with these studies. Our study only includes 38% of SGLT2i prescription at the end of follow-up, whose use was limited because its indication in patients with HFrEF appeared in the ESC 2021 guidelines.

Data related to ventricular remodelling were assessed only by echocardiography; insufficient data on global longitudinal strain and cardiac magnetic resonance imaging are available. In addition, no data were available regarding the number of visits at follow-up or the assessment of symptoms using the Kansas City Cardiomyopathy or Minnesota questionnaire.

Another limitation is the exclusion of advanced HF cases receiving LVAD and transplantation, which were excluded for assessment of ventricular remodelling and function at follow-up.

## 5. Conclusions

Patients with ≥2 previous admissions for HF have a longer disease course and a higher proportion of comorbidities such as atrial fibrillation, anaemia and CKD. These patients seem to have a worse medium- to long-term prognosis in terms of higher rates of hospital readmissions and cardiovascular mortality from early stages, so it is essential to identify them early for closer follow-up and the application of more intensive pharmacological treatment that includes new therapeutic options with prognostic benefit in this high-risk population.

## Figures and Tables

**Table 1:** Baseline clinical characteristics. BMI: body mass index; CKD: chronic kidney disease; Cr: creatinine; DBP: diastolic blood pressure; DLP: dyslipidaemia; DM: diabetes mellitus; GFR: glomerular filtration rate; Hb: haemoglobin; HF: heart failure; HFrEF: heart failure with reduced ejection fraction; HR: heart rate; HT: hypertension; NYHA: New York Heart Association classification; SBP: systolic blood pressure; TSI: transferrin saturation Index.

	HFrEF (N = 406)	Prior HF Admission: 0–1 (N = 361)	Prior HF Admission: ≥2 (N = 45)	*p*
Male sex (%)	300 (73.9%)	265 (73.4%)	35 (77.8%)	0.529
Age (year)	69.0 (59–77)	68.0 (59–77)	72.0 (64–77)	0.069
CV risk factors				
DM	195 (48.0%)	168 (46.5%)	27 (60.0%)	0.088
HT	273 (67.2%)	237 (65.7%)	36 (80.0%)	0.053
DLP	252 (62.2%)	212 (58.9%)	40 (88.9%)	<0.001
Smoking	50 (13.7%)	40 (12.3%)	10 (23.8%)	
Former smoker	162 (44.3%)	147 (45.4%)	15 (35.7%)	0.118
HF de novo	226 (55.7%)	226 (62.6%)	0 (0%)	<0.001
Heart failure evolution time (months)	36.3 ± 66.7	28.0 ± 60.3	103.6 ± 77.9	<0.001
LVEF improvement	156 (41.9%)	154 (46.7%)	2 (4.8%)	<0.001
NYHA				
I	61(15.1%)	59 (16.4%)	2 (4.4%)	
II	265 (65.4%)	232 (64.4%)	33 (73.3%)	0.201
III–IV	79 (19.9%)	69 (19.2%)	10 (22.2%)	
Aetiology				
Ischaemic	131 (32.3%)	111 (30.7%)	20 (44.4%)	0.064
Valvular	6 (1.5%)	6 (1.7%)	0 (0%)	1.000
Tachycardiomyopathy	44 (10.8%)	44 (12.2%)	0 (0%)	0.009
Toxic	15 (3.7%)	12 (3.3%)	3 (6.7%)	0.225
Cardiotoxicity	16 (3.9%)	16 (4.4%)	0 (0%)	0.235
Genetic	12 (3.0%)	10 (2.8%)	2 (4.4%)	0.631
Idiopathic	125 (30.8%)	114 (31.6%)	11 (24.4%)	0.262
Atrial Fibrillation	213 (52.5%)	180 (49.9%)	33 (73.3%)	0.003
BMI > 30	145 (37.4%)	126 (36.7%)	19 (42.2%)	0.636
Baseline HR (beats/min)	71.0 (62–81)	71.0 (62–82)	71.0 (63–78)	0.824
Baseline SBP (mm Hg)	110.0 (100.0–122.8)	110.0 (99.8–121.3)	111.0 (98.9–129.3)	0.523
Baseline DBP (mm Hg)	69.0 (60–77)	69.0 (60.3–77.8)	65.0 (60–70.5)	0.13
QRS width (ms)	109.5 (88.8–135)	108 (88.5–135)	122.0 (93–149)	0.267
QRS ≥ 120 ms	149 (40.4%)	125 (37.8%)	24 (63.2%)	0.003
BCRD	33 (9.4%)	24 (7.5%)	9 (25.0%)	0.003
BCRI	115 (32.7%)	100 (31.6%)	15 (41.7%)	
CKD	168 (41.4%)	131 (36.3%)	37 (82.2%)	<0.001
3a	55 (13.6%)	47 (13.0%)	8 (17.8%)	
3b	66 (16.3%)	51 (14.1%)	15 (33.3%)	
IV	33 (8.1%)	21 (5.8%)	12 (26.7%)	<0.001
V	14 (3.5%)	12 (3.3%)	2 (4.4%)	
Cr (mg/dL)	1.1 (0.9–1.4)	1.0 (0.9–1.3)	1.6 (1.1–1.8)	<0.001
GFR (mL/min)	68.0 (48–86)	70.0 (50.5–87.5)	43 (32.5–69)	<0.001
Anaemia (%)	128 (31.5%)	104 (28.8%)	24 (53.3%)	0.001
Hb (g/dL)	13.7 (12.1–15.2)	13.9 (12.2–15.3)	13.2 (11.4–14.1)	0.023
Ferritin (ug/L)	99.3 (49.6–268.4)	99.0 (50.1–270.5)	101.7 (44.4–244)	0.943
TSI (%)	17.3 (11.7–27.7)	16.5 (11.3–27.5)	20.9 (14.4–29)	0.149
Sodium (mEq/L)	140.0 (137–142)	140.0 (137–142)	139.0 (137–141)	0.368
Potassium (mEq/L)	4.4 (4–4.7)	4.4 (4–4.7)	4.2 (3.6–4.7)	0.030
CA-125 (U/mL)	21.8 (9.5–69)	19.8 (9.4–65.8)	66 (11.4–149.5)	0.177
NT-proBNP (pg/mL)	4848 (2084.5–11,009)	4640.0 (1873–10,431)	6297.0 (3981.8–18,075.8)	0.005
Glycated Hb (%)	6.3 (5.8–7.1)	6.2 (5.8–7.1)	6.7 (6–7.2)	0.117

**Table 2:** Clinical, analytical, echocardiographic, and treatment parameters at baseline and at the end of follow-up. ACEI: angiotensin-converting enzyme inhibitors; ARB: angiotensin receptor blockers; ARNI: angiotensin and neprilysin receptor inhibitors; Cr: creatinine; CRT: cardiac resynchronisation therapy; DBP: diastolic blood pressure; GFR: glomerular filtration rate; Hb: haemoglobin; HF: heart failure; HFrEF: heart failure with reduced ejection fraction; HR: heart rate; ICD: implantable cardioverter defibrillator; LVEDD: left ventricular end-diastolic diameter; LVEDV: left ventricular end-diastolic volume; LVEF: left ventricular ejection fraction; LVESD: left ventricular end-systolic diameter; LVESV: left ventricular end-systolic volume; MR: mitral regurgitation; MRA: mineralocorticoid receptor antagonist; NT-proBNP: N-terminal pro-brain natriuretic peptide; NYHA: New York Heart Association classification; PMVT: percutaneous mitral valve treatment; SBP: systolic blood pressure; SGLT2i: sodium glucose cotransporter type 2 inhibitor. TSI: transferrin saturation index.

	HFrEF (N = 406)			Prior HF Admission: 0–1 (N = 361)			Prior HF Admission: ≥2 (N = 45)		
	Baseline	End of Follow-Up	*p*	Baseline	End of Follow-Up	*p*	Baseline	End of Follow-Up	*p*
LVEF (%)	30.0 (26–35)	38.0 (30–52)	<0.001	30.0 (26–35)	40.0 (31–53.5)	<0.001	30.0 (26.5–35)	30.0 (26–35)	0.994
LVEDD (mm)	62.0 (57–68)	58.0 (53–63)	<0.001	61.0 (57–67)	58.0 (52–62.5)	<0.001	67.0 (58.8–70.3)	60.0 (56.5–64.5)	0.010
LVESD (mm)	52.0 (47–59)	45.0 (38–52)	<0.001	51.0 (47–59)	43.0 (37–51.3)	<0.001	54.0 (49.5–60)	50.5 (48–58.3)	0.410
LVEDV (mL)	153 (117.8–185.5)	124.0 (91–166)	<0.001	150.0 (117–183)	118 (87.5–164)	<0.001	176.0 (146–224)	160.5 (120.8–196.5)	0.794
LVESV (mL)	104 (78–136)	72.5 (47–107.3)	<0.001	101.0 (78–130.8)	69 (45–105.3)	<0.001	119.0 (101–158)	109.5 (90.3–131.8)	0.469
Severe MR (%)	44 (11%)	21 (5.8%)	<0.05	36 (10.1%)	15 (4.6%)	<0.05	8 (17.8%)	6 (14.3%)	1.000
HR (bpm)	71.0 (62–81)	71.0 (63–82)	0.169	71.0 (62–82)	71.0 (62–82)	0.228	71.0 (63–78)	71.0 (68.5–83.5)	0.466
SBP (mm Hg)	110.0 (100.0–122.8)	117.0 (102–130)	0.001	110.0 (99.8–121.3)	117.0 (102–130)	0.002	111.0 (98.9–129.3)	114.0 (102.3–129.3)	0.160
DBP (mm Hg)	69.0 (60–77)	70.0 (60–80)	0.181	69.0 (60.3–77.8)	70.0 (60–80)	0.402	65.0 (60–70.5)	70.0 (60–75)	0.069
NYHA III–IV	79 (19.9%)	84 (20.7%)	0.675	69 (19.2%)	57 (15.8%)	0.189	10 (22.2%)	27 (60.0%)	<0.001
NT-proBNP (pg/mL)	4848 (2084.5–11,009)	1931 (608–6282)	<0.001	4640.0 (1873–10,431)	1599 (522.8–5148.5)	<0.001	6297.0 (3981.8–18,075.8)	8136 (3407–16,339)	0.961
CA-125 (U/mL)	21.8 (9.5–69)	10.1 (6.2–21)	0.016	19.8 (9.4–65.8)	10.1 (6.2–20.9)	0.011	66 (11.4–149.5)	10.5 (7.7–62.5)	0.917
Glycated Hb (%)	6.3 (5.8–7.1)	6.2 (5.8–6.9)	<0.001	6.2 (5.8–7.1)	6.2 (5.7–6.9)	<0.001	6.7 (6–7.2)	6.6 (5.8–7.8)	0.703
Hb (g/dL)	13.7 (12.1–15.2)	13.6 (12.1–15.3)	0.446	13.9 (12.2–15.3)	13.7 (12.1–15.4)	0.675	13.2 (11.4–14.1)	13.0 (11–14.3)	0.283
Cr (mg/dL)	1.1 (0.9–1.4)	1.2 (1.0–1.6)	<0.001	1.0 (0.9–1.3)	1.1 (1.0–1.5)	<0.001	1.6 (1.1–1.8)	1.6 (1.4–2.2)	0.001
GFR (mL/min)	68.0 (48–86)	59.0 (40–78)	<0.001	70.0 (50.5–87.5)	62.0 (43–80)	<0.001	43 (32.5–69)	39 (28–51.5)	0.002
Ferritin (ug/L)	99.3 (49.6–268.4)	93.2 (43–247.2)	0.383	99.0 (50.1–270.5)	90.9 (43–230)	0.192	101.7 (44.4–244)	104.9 (39–387.3)	0.322
TSI (%)	17.3 (11.7–27.7)	21.5 (13.8–30.8)	0.027	16.5 (11.3–27.5)	21.8 (13.9–31.5)	0.023	20.9 (14.4–29)	18.0 (10.4–23.6)	1.000
ACEI/ARB (%)	233 (57.4%)	127 (31.3%)	<0.001	214 (59.3%)	118 (32.7%)	<0.001	19 (42.2%)	9 (20.0%)	0.013
ARNI (%)	139 (34.2%)	241 (59.4%)	<0.001	120 (33.2%)	214 (59.3%)	<0.001	19 (42.2%)	27 (60.0%)	0.057
Beta-blockers (%)	371 (91.4%)	355 (87.4%)	0.021	329 (91.1%)	315 (87.3%)	<0.05	42 (93.3%)	40 (88.9%)	0.668
Loop diuretics (%)	326 (80.3%)	291 (71.7%)	0.001	283 (78.4%)	248 (68.7%)	0.001	43 (95.6%)	43 (95.6%)	1.000
Thiazides (%)	75 (18.5%)	77 (19.0%)	1.000	59 (16.3%)	56 (15.5%)	0.807	16 (35.6%)	21 (46.7%)	0.267
MRA (%)	299 (73.6%)	283 (69.7%)	0.133	264 (73.1%)	249 (69.0%)	0.120	35 (77.8%)	34 (75.6%)	1.000
Ivabradine (%)	88 (21.7%)	77 (19.0%)	0.194	81 (22.4%)	72 (19.9%)	0.222	7 (15.6%)	5 (11.7%)	0.625
Digoxin (%)	48 (11.8%)	49 (12.1%)	1.000	40 (11.1%)	40 (11.1%)	1.00	8 (17.8%)	9 (20.0%)	1.000
SGLT2i (%)	75 (18.5%)	153 (37.7%)	<0.001	67 (18.6%)	139 (38.5%)	<0.001	8 (17.8%)	14 (31.1%)	0.109
CRT (%)	19 (4.7%)	68 (16.7%)	<0.001	9 (2.5%)	53 (14.7%)	<0.001	10 (22.2%)	15 (33.3%)	0.063
ICD (%)	35 (8.6%)	71 (17.5%)	<0.001	25 (6.9%)	55 (15.2%)	<0.001	10 (22.2%)	16 (35.6%)	0.031
PMVT (%)	1 (0.2%)	15 (3.7%)	<0.001	1 (0.3%)	11 (3%)	<0.05	0 (0%)	4 (8.9%)	0.125

**Table 3:** Admission for heart failure and cardiovascular mortality. Cox univariate and multivariate model. LBBB: left bundle branch block. LVEF: left ventricular ejection fraction. NYHA: New York Heart Association functional class.

	Admission for Heart Failure Univariate and Multivariate Cox Model						Cardiovascular Mortality Univariate and Multivariate Cox Model					
	Univariate			Multivariate			Univariate			Multivariate		
	HR ^1^	95% CI ^1^	*p*-Value	HR ^1^	95% CI ^1^	*p*-Value	HR ^1^	95% CI ^1^	*p*-Value	HR ^1^	95% CI ^1^	*p*-Value
Female sex	1.26	0.88, 1.80	0.200	1.16	0.78, 1.71	0.467	1.08	0.67, 1.73	0.755	0.92	0.54, 1.54	0.745
Age	1.04	1.02, 1.05	<0.001	1.00	0.98, 1.01	0.736	1.06	1.04, 1.08	<0.001	1.04	1.01, 1.06	0.004
>1 prior admissions HF	3.34	2.28, 4.89	<0.001	2.11	1.39, 3.21	<0.001	4.47	2.84, 7.02	<0.001	2.84	1.75, 4.59	<0.001
Diabetes mellitus	1.25	0.90, 1.71	0.179	0.71	0.49, 1.02	0.066	1.21	0.80, 1.83	0.362			
Hypertension	1.97	1.35, 2.89	<0.001	1.47	0.97, 2.25	0.071	1.83	1.11, 3.01	0.017			
Dyslipidaemia	1.68	1.18, 2.38	0.004				2.11	1.30, 3.41	0.002			
Smoking	0.95	0.69, 1.31	0.745				0.85	0.56, 1.29	0.448			
Atrial fibrillation	1.58	1.13, 2.19	0.007	1.22	0.85, 1.75	0.273	1.55	1.02, 2.37	0.042			
Chronic kidney disease	4.12	2.92, 5.80	<0.001	2.52	1.66, 3.82	<0.001	5.11	3.20, 8.16	<0.001	2.38	1.39, 4.06	0.001
Obesity	0.73	0.60, 0.90	0.002	0.71	0.57, 0.88	0.002	0.75	0.58, 0.97	0.030			
Anaemia	3.78	2.74, 5.22	<0.001	1.86	1.27, 2.74	0.002	4.78	3.11, 7.33	<0.001	2.01	1.23, 3.28	0.005
Ischaemic aetiology	1.65	1.19, 2.29	0.003	1.28	0.87, 1.89	0.209	2.02	1.33, 3.06	<0.001	1.30	0.83, 2.05	0.256
Baseline LVEF	1.01	0.98, 1.03	0.662	0.99	0.96, 1.02	0.576	1.00	0.97, 1.04	0.997	0.98	0.94, 1.01	0.175
NYHA III–IV	2.42	1.70, 3.43	<0.001	2.18	1.50, 3.17	<0.001	2.59	1.68, 4.02	<0.001	2.54	1.61, 4.02	<0.001
LBBB	1.56	1.13, 2.16	0.007	1.14	0.79, 1.63	0.482	1.58	1.04, 2.41	0.032			

^1^ HR = hazard ratio, CI = confidence interval.

## Data Availability

The data presented in this study are available upon request from the corresponding author.

## Supplementary Materials

The following supporting information can be downloaded at: https://www.mdpi.com/article/10.3390/jcm12186082/s1, Figure S1: Flow chart of the study; Table S1: Main cardiovascular and no-cardiovascular mortality data.
