# Supplementary material for: The Worsening of Heart Failure with Reduced Ejection Fraction: The Impact of the Number of Hospital Admissions in a Cohort of Patients
Source: J Clin Med. 2023 Sep 20;12(18):6082. doi: 10.3390/jcm12186082 (PMC10531712; doi:10.3390/jcm12186082)
Supplement: Supplementary file 1 [file jcm-12-06082-s001.zip › jcm-2585965-supplementary.pdf]

SUPPLEMENT METHODS

1. Flow chart

In this section, we include the flow chart of the study. In our study, we included 422 patients with a diagnosis of HFrEF from 2018 to 2020 and the follow-up was until November 2022. In the follow-up, we excluded 3 patients because we did not have data on previous admissions as they came from other centres and we excluded 13 patients who received a heart transplant in the follow-up.

At this point, we divided the patients into two groups according to previous admissions for HF: 0-1 previous admissions for HF (n=361) and ≥2 previous admissions for HF (n=45).

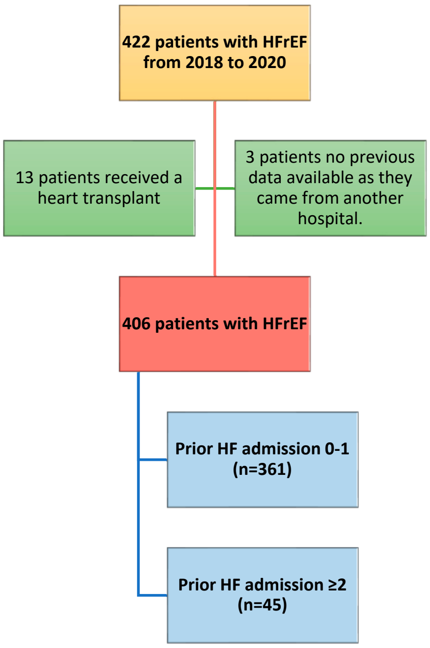

Figure S1. Flow chart of the study.

2. Methods utilized in order to identify heart failure etiology.

All patients underwent transthoracic echocardiography and different tests to rule out ischaemic aetiology (magnetic resonance imaging, coronary angio-CT and/or coronary angiography).

In the absence of coronary artery disease justifying the dysfunction, different tests were performed depending on the clinical context (mainly magnetic resonance imaging if not already performed). In the absence of a clear triggering factor (tachycardiomyopathy,

valvular heart disease, alcohol, cardiotoxicity…), a genetic study was performed. Finally, in the absence of the other aetiologies and of a clearly pathogenic mutation related to the patient's clinical manifestation, idiopathic aetiology was considered.

3. Definition of cardiovascular and non cardiovascular causes of death.

Cardiovascular death is defined as death due to pump failure, acute myocardial infarction and sudden death. Sudden death was considered to be all deaths occurring unexpectedly and unwitnessed in the absence of another concomitant active pathological process (active infection, non-curable neoplastic cancer…). Death from heart failure included pump failure and sudden death. Any death that did not meet the previously established criteria was considered a non-cardiovascular death.

SUPPLEMENT RESULTS

1. Results on cardiovascular and non-cardiovascular mortality.

In our study, there was a trend towards a higher proportion of non-cardiovascular deaths in the Prior HF admission 0-1 group compared to the Prior HF admission ≥2 group, but without reaching statistically significant differences (p=0.111).

Table S1. Main cardiovascular and no-cardiovascular mortality data.

	**Prior HF admission: 0-1 (N=92)**	**Prior HF admission: ≥2 (N=31)**
**Pump failure**	45 (48.9%)	19 (61.3%)
**Myocardial infarction**	0 (0%)	0 (0%)
**Sudden death**	18 (19.4%)	8 (25.8%)
**No cardiovascular cause**	29 (31.5%)	4 (12.9%)

2. Results on intravenous administration of carboxymaltose.

In our study, data were collected on the need for intravenous administration of carboxymaltose at some point during follow-up. There was no statistically significant difference in intravenous administration of carboxymaltose between the two groups:

Prior HF admission 0-1 (n=63) and Prior HF admission ≥2 (n=10) (17.5% vs 22.2%; p=0.432).
